# 72 revision surgeries for aseptic failure after hip or knee arthroplasty: a prospective study with an extended diagnostic algorithm

**DOI:** 10.1186/s12891-019-2944-y

**Published:** 2019-12-12

**Authors:** Vesal Khalid, Henrik Carl Schønheyder, Poul Torben Nielsen, Andreas Kappel, Trine Rolighed Thomsen, Ramune Aleksyniene, Jan Lorenzen, Sten Rasmussen, The PRIS Study group, Lone Heimann Larsen, Ole Simonsen, Camilla Rams Rathleff, Line Rode Abrahamsen, Ulla Hornum, Sanne Riss, Hanne Brink, Mogens Brouw Jørgensen, Mogens Berg Laursen, Christian Pedersen, Jess Riss, Yijuan Xu, Lars Arendt-Nielsen, Kristian Kjær Pedersen, Morten Karsdal, Jeppe Lange

**Affiliations:** 1 Orthopaedic Research Unit, Aalborg University Hospital, Aalborg, Denmark; 2 Department of Orthopaedic Surgery, Aalborg University Hospital, Aalborg, Denmark; 3 Department of Clinical Medicine, Aalborg University, Aalborg, Denmark; 4 Department of Clinical Microbiology, Aalborg University Hospital, Aalborg, Denmark; 5 Center for Microbial Communities, Department of Biotechnology, Chemistry and Environmental Engineering, Aalborg University, Aalborg, Denmark; 6 Danish Technological Institute, Medical Biotechnology, Aarhus, Denmark; 7 Department of Nuclear Medicine, Aalborg University Hospital, Aalborg, Denmark

## Abstract

**Background:**

Unrecognized periprosthetic joint infections are a concern in revision surgery for aseptic failure (AF) after total hip (THA) or knee (TKA) arthroplasties. A gold diagnostic standard does not exist. The aim of the current study was to determine the prevalence of unrecognized periprosthetic joint infection (PJI) in a cohort of revision for AF, using an experimental diagnostic algorithm.

**Methods:**

The surgeons’ suspicion of AF was based primarily on patient history and clinical evaluation. X-ray imaging was used to reveal mechanical problems. To rule out an infectious aetiology standard blood biochemical tests were ordered in most patients. Evaluation followed the existing practice in the institute. Cases were included if revision surgery was planned for suspected AF. Intraoperatively, five synovial tissue biopsies were obtained routinely. PJI was defined as ≥3 positive cultures with the same microorganism(s). Patients were followed for 1 year postoperatively. Protocol samples included joint fluid, additional synovial tissue biopsies, bone biopsy, swabs from the implant surface, and sonication of retrieved components. Routine and protocol samples were cultured with extended incubation (14 days) and preserved for batchwise 16S *rRNA* gene amplification. Patients were stratified based on culture results and a clinical status was obtained at study end.

**Results:**

A total of 72 revisions were performed on 71 patients (35 THA and 37 TKA). We found five of 72 cases of unrecognized PJI. Extended culture and protocol samples accounted for two of these. One patient diagnosed with AF was treated for a PJI during follow-up. The remaining patients did not change status from AF during follow-up.

**Conclusions:**

We found a low prevalence of unrecognized periprosthetic joint infections in patients with an AF diagnosis. The algorithm strengthens the surgeons’ preoperative diagnosis of a non-infective condition. Evaluation for a failing TKA or THA is complex. Distinguishing between AF and PJI pre-operatively was a clinical decision. Our data did not support additional testing in routine revision surgery for AF.

## Introduction

The number of patients with failing total hip (THA) or knee (TKA) arthroplasties is rising due to the wide access to primary arthroplasties and the increasing longevity of patients [1, 2]. Scandinavian arthroplasty registries report aseptic failure (AF) as the most common indication for revision surgery followed by periprosthetic joint infection (PJI) [3, 4]. AF is a collective term for aseptic conditions, most of which are implant loosening, instability, and polyethylene wear [5].

It remains controversial whether some cases of AF may indeed be caused by low-grade infection associated with microorganisms forming biofilms [6–8]. Biofilm-associated microorganisms are able to evade the immune response and thus typically produce a poor local and systemic host reaction [9].

At the present time, no gold standard exists for diagnosis of PJI. Pre-operative diagnostics include patient history, clinical examination, X-ray imaging, blood biochemistry, and synovial fluid analysis by microscopy and culture [10]. Intra-operatively macroscopic findings are essential and various samples can be obtained for definitive diagnosis. For several decades culturing of synovial tissue biopsies from the vicinity of the implant has been a mainstay for ruling out PJI [11–13]. Several institutes define PJI as 2 positive periprosthetic cultures with phenotypically identical organisms [14, 15]. However no consensus exists. The concern for biofilm-forming bacteria has brought attention to the implant as the nidus of infection, and samples can be prepared in the laboratory by sonication of removed prosthesis parts [16]. New diagnostic options have been provided by modification of well-established culture methods and a range of DNA-based techniques.

This development has led to a number of studies critically examining the confirmation of AF [17–21]. There may be reservations when interpreting such studies especially concerning the design, selection of patients, compliance with a strict protocol, standardization, and availability of novel techniques. Head-to-head comparisons of different methods have rarely been possible and their diagnostic accuracy remains debatable [22].

With an extensive diagnostic work-up, it may be possible with greater certainty to determine the prevalence of unrecognized PJI infection in patients undergoing revision for AF. The aim was to characterize AF patients with a range of supplementary microbiological diagnostic methods applied to standard and experimental specimen types obtained intraoperatively.

## Materials and methods

Patients were invited to participate in a study with the primary aim to improve the diagnosis of prosthesis-related infection and pain (Danish acronym PRIS) through a multidisciplinary diagnostic algorithm [23]. The project was carried out between 2011 and 2014 in the North Denmark Region, with a population of approximately 580.000.

### The inclusion of patients in the PRIS project

Patients referred to the Department of Orthopaedic Surgery, Aalborg University Hospital by general practitioners or other hospital departments were included prospectively from December 2011 to January 2014. Inclusion criteria in the main project from which this study arises were a prosthetic failure and/or suspected infection. Based on history and clinical examination, failure was defined as unexplained pain and/or a mechanical problem (loosening or wear revealed by X-ray imaging and clinical judgment). Infection was suspected in presence of a communicating sinus, or an acutely unwell patient with fever and a swollen joint compatible with a haematogenous infection.

Exclusion criteria were repeated dislocation (in THA), age below 18 years, or fracture.

The current study was confined to patients undergoing revision surgery on suspicion of AF defined as a failure and an infectious aetiology being deemed unlikely judged clinically by a senior orthopaedic surgeon.

Aseptic failure was suspected by the presence of pain during activities and the radiological presence of radiolucency and/or component migration. In TKA mal-positioning and instability was judged clinically and supported by radiological examinations. In THA radiological examination was assessed for implant migration and radiolucency around the implant. PJI was suspected in the presence of pain, redness, swelling, secretion from the joint and/or elevated inflammatory markers (CRP and WBC). A local document with guidelines and instructions was followed if there was a suspicion of PJI.

A pragmatic study design with pre-operative evaluation was chosen in order to follow the existing practice in the department. In every case the surgeon obtained the history and examined the patient. Ordering X-ray examination and routine inflammatory markers (leukocyte count in peripheral blood and plasma C-reactive protein) were on the surgeon’s discretion.

Joint aspiration was discouraged as a primary diagnostic procedure due to possible interference with subsequent multimodal nuclear imaging [24]. If clinical suspicion of PJI was present or further evaluation was initiated, patients continued the investigation in the algorithm (see Appendix 1). The main differences from the standard procedure was an option for nuclear imaging and an extended protocol for diagnostic sampling during revision surgery (referred to as project samples and tests) [25].

### Revision surgery

Surgical revision followed the department’s routines including a sampling of five periprosthetic synovial tissue biopsies taken with separate instruments according to Kamme and Lindberg [11]. According to the protocol triplicate samples of joint fluid, periprosthetic synovial tissue, periprosthetic bone tissue, and swabs from the implant surface were taken along with any exchanged prosthetic components (Appendix 2). Joint fluid was aspirated with a needle once the joint was exposed, but prior to incision of the capsule. Next protocol samples were taken from the vicinity of the prosthesis. To support these elaborate procedures all containers and transport media were provided as a set in a box [25]. Transport and handling of specimens including sonication of prosthetic components have been described elsewhere [23, 25]. Triplicates made it feasible to perform parallel testing by bacteriological culturing for 14 days (routine 6 days) and 16S *rRNA* gene amplification followed by amplicon sequencing (22). In total 4 protocol samples and 5 standard periprosthetic synovial tissue biopsies were cultured. The molecular tests were carried out batch-wise and the results did not influence clinical management due to the delay. Final culture reports from standard tissue biopsies were available after 6 days as usual. However, surgeons were notified about late positive cultures (i.e. after day 6) if they deviated from the standard cultures.

### Clinical follow-up

Clinical follow-up was defined by appointment with the surgeon at 6 weeks and 1 year postoperatively. Medical records and a laboratory database were reviewed in August 2015 for all contacts with the Department of Orthopaedic Surgery and any microbiological samples of relevance from health services in the region.

### Data sources

Patients in Denmark have a unique identification number enabling control of missing data at inclusion and follow-up. Baseline characteristics of patients, comorbidities, previous history of the affected joint and prior antibiotic treatment to revision surgery were obtained. Supplementary data were obtained from medical records and the Danish Hip and Knee Arthroplasty Registries (DHR and DKR) where indication for surgery are routinely notified.

Blood biochemistry values were obtained from the laboratory information system (Labka, CSC, Denmark): C-reactive protein (CRP) (normal range ≤ 8 mg/L); white blood cell (WBC) (normal range: count 3.5–10.0 × 10^9^/L).

### Data analysis

Patients were divided into three groups on the basis of culture reports from the 5 routinely obtained periprosthetic synovial tissue biopsies: Negative cultures (Group 1), one or two positive cultures (Group 2), and three to five positive cultures (Group 3). The cut-off point of three positive cultures for PJI followed the recommendation by Kamme & Lindberg and has been a diagnostic criterion for PJI since the mid-1990s [11]. Descriptive characteristics were reported for these three groups.

Data are presented in median and interquartile range. Mann-Whitney test are used for comparison between groups. Logistic regression was used to determine any independent variables for no positive periprosthetic biopsies. The statistical program Stata/MP 15.1 was used.

Approval of the PRIS project was obtained from the Regional Committee on Health and Research Ethics for the Northern Denmark Region (N-20110022) and the Danish Data Protection Agency (2008-58-0028).

## Results

**Fig. 1:**
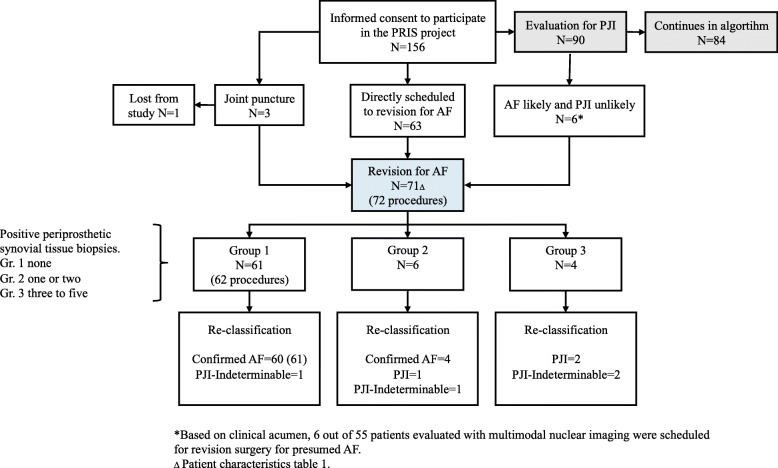
Flowchart

During the study period, 156 patients were included. Seventy-two revisions were performed on the indication of AF in 71 patients (35 THA and 37 TKA; flow is depicted in Fig. 1. Data for the remaining 85 included on the suspicion of PJI or pain not shown). Patient characteristics are shown in Table 1. A re-classification was based on the laboratory work on the project samples: Confirmed AF, PJI and PJI-indeterminable status (Table 2).

**Table 1:** Demographics and characteristics of 71 patients undergoing revision surgery for suspected aseptic failure of a hip or knee arthroplasty

Presumed aseptic failure (*n* = 71^b^)	
Age, years (mean, SD)	70 (12.5)
Sex, number (females)	40
Comorbidities (*n*)	
Rheumatic disease	4
Cardiovascular disease	14
Diabetes mellitus	7
Cancer	6
COPD	4
Biological immunotherapy	2
BMI (mean, SD)	29.9 (5.0)
Joint	
Hip	35
Knee	36
Prosthesis, age (*n*)	
≤ 30 days	1
31–365 days	8
1–5 years	21
6–10 years	9
11–15 years	11
≥ 15 years	19
Indication for previous surgery (*n*)	
Aseptic failure	7
Prosthetic joint infection	1
Symptoms and signs at inclusion (*n*)^a^	
Pain	67
Reduced mobility	38
Swelling	4
Redness	2
Warmth	2

Diabetes mellitus: type 1 and type 2; Cardiovascular disease: Hypertension, ischaemic heart disease, COPD: Chronic Obstructive Pulmonary Disease, Cancer: All types except skin cancer treated within the last 5 years; Biological Immunotherapy

^a^Surgeon’s elicited symptoms

^b^Patient with revision on two different occasions is featured with the first

**Table 2:** Findings in 72 revisions of hip and knee arthroplasties on indication of aseptic failure. The groups are defined by results obtained with the reference method, i.e. culture of five periprosthetic synovial tissue biopsies from each procedure. Gr. 1: 0 positive, Gr. 2–3 positive. Gr. 3: 3–5 positive

	Group 1	Group 2	Group 3
No. of revisions (and patients)	62 (61)	6	4
Arthroplasties (no.)	THA 29; TKA 33	THA 3; TKA 3	THA 3; TKA1
Prosthesis age, years (median, interquartile range (0.25–0.75))	9.2 (2.7–15.8)	11.8 (3.1–19.9)	7.6 (5.5–9.2)
Prior antibiotic treatment (no.)	3	0	0
CRP (μg/mL, (median, interquartile range (0.25–0.75))	7.4 (1.5–8.5)^c^	6.5 (1–2.5)^c^	28.2 (20.3–39.5)^c^
WBC (range 10^9^/L, (median, interquartile range (0.25–0.75))	7.2 (6.1–8.4)	5.9 (3.9–6.8)	7.5 (6.4–9.1)
Number of positive periprosthetic synovial tissue biopsies (a set comprises 5 biopsies)	0	1–2	3–5
Reclassification based on work-up of standard tissue samples			
Aseptic failure	61	4	0
Prosthetic joint infection	0	1^a^	4^a^
Indeterminable	1^a^	1^a^	0
Project diagnostics, culture (Cul) and molecular (Mol), number of cases			
Joint fluid	Cul 2 Mol 1	Cul 2 Mol 0	Cul 3 Mol 1
Sonication fluid	Cul 2 Mol 0	Cul 1 Mol 1	Cul 3 Mol 3
Bone biopsy	Cul 0 Mol 2	─	Cul 1 Mol 0
Swab from prosthetic element	Cul 1 Mol 3	─	Cul 2 Mol 1
Follow-up period, days (median & range)	396 (3^b^-1097)	367 (47–1131)	363 (96–1051)
Indication for revision during follow-up			
Aseptic failure	4	0	0
Prosthetic joint infection	1	0	0

^a^Case 1–6 and 11 in Appendix 3

^b^Patient died from cardiac arrest during admittance

^c^Missing values: Group 1: 11; Group 2: 1; Group 3: 1

At inclusion, 64 patients had pain as the dominant symptom followed by reduced mobility (Table 1). Four patients had an indolent prosthetic joint had joint swelling and reduced mobility. Two patients had a preoperative joint aspiration at the discretion of the surgeon. Culturing and molecular tests were negative in both cases. Six patients were scheduled for revision surgery following multi-modal nuclear imaging which showed no pathological uptake on dual white blood cell (WBC)/bone marrow scans in all the cases (Fig. 1). Fluor-deoxy-glucose (FDG) PET/CT scans showed FDG uptake in the periprosthetic synovial tissue or in the interface between bone and implant in five out of the six cases, compatible with both PJI and AF. Clinical acumen supported revision for AF.

### Samples

Completeness of synovial tissue biopsies for culture was 100%. Completeness of project samples was 89.9 and 86.1% for culture and molecular analysis, respectively. Implant components for sonication were retrieved in 67 cases. A total implant replacement was made in 14 cases, and a single or multiple components were exchanged in the remaining cases, Table 3. The intraoperative clinical inspection did not conflict with the preoperative indication in any case.

**Table 3:** Indication and information regarding exchange of components during revision surgery of THA or TKA. Ortopaedic surgeons notify routinely the two Danish National Arthroplasty Registries for hip or knee arthroplasties. If information was missing the medical record was an alternative source

THA	
Aseptic failure of	
Femur and acetabulum component with osteolysis	4
Femur component with osteolysis	9
Femur component without osteolysis	4
Acetabulum component with osteolysis	9
Acetabulum component without osteolysis	5
Supracondylar femoral fracture	2
Other	2
Components exchanged	
Total replacement	4
Single components	
Acetabulum component	13
Acetabulum liner	16
Caput	23
Femur component	12
Soft tissue revision without exchange of prosthesis	1
TKA	
Aseptic failure of	
Tibia component	17
Tibia and femoral component	3
Femur component	1
Tibia polyethylene	5
Instability	5
Other	5
Components exchanged	
Total replacement	10
Single components	
Tibia component	23
Femur component	15
Patella component	2
Soft tissue revision without exchange of prosthesis	1

Positive cultures obtained by prolonged incubation of synovial tissue biopsies (*n* = 10) became available after a median period of 8 days with an upper range of 15 days.

Key findings for the entire study group are summarized in Table 2. Logistic regression using CRP, WBC, sex, age BMI and co-morbidity found young age (*P* = 0.013) and normal BMI (< 25) (*P* = 0.013) was associated with not having positive periprosthetic synovial tissue biopsy cultures. No differerences in CRP and WBC was found between the groups, except for CRP between group 1 and group 3 (*P* = 0.043).

Results from standard and project samples are summarized for 19 patients in Appendix 3.

A patient with bilateral TKA in Group 1 (*n* = 61) underwent revision on two different occasions in separate joints, respectively. CRP was within normal range in 37 patients, and elevated in 13 cases. In three patients antibiotics had been administered within 4 weeks of surgery for an unrelated cause, and cultures of standard and protocol samples were negative.

One patient (case 11) was re-classified as PJI-indeterminable. During revision, a large subfascial accumulation of serous fluid flooded the surgical field, and the finding of *Escherichia coli* by the culture of joint fluid and sonication fluid was an uncertain finding.

During the follow-up, five revisions were recorded in group 1, one for PJI and four for AF. The patient with PJI underwent a second revision 825 days later outside the PRIS project. The first revision within the PRIS project revealed negative project samples by both culture and molecular tests. The second revision during follow-up revealed *Enterococcus faecalis* in periprosthetic synovial tissue biopsies (5/5). This finding was deemed unrelated to the condition which prompted the previous revision. The intraoperative sets of periprosthetic synovial tissue biopsies from the four patients with AF were negative by standard culture for 6 days.

In Group 2 (*n* = 6) CRP was not elevated in four and elevated in one patient (one had missing values). One patient was re-classified as a PJI (case 5) on the basis of two periprosthetic synovial tissue biopsies and joint culture with *Propionibacterium acnes.* Severe inflammation without pus was observed intraoperatively. Review of medical records revealed an elevation of CRP (> 100 mg/L) in connection with medical treatment for an unrelated condition 2 months previously.

Another patient was re-classified as PJI-indeterminable (case 6): One periprosthetic synovial tissue biopsy and joint fluid culture revealed *P. acnes* after culture for 14 days of incubation. Intraoperatively, moderate inflammation was observed.

The four patients in Group 3 were re-classified to PJI. They all had 3–5 positive periprosthetic synovial tissue cultures. CRP was elevated in two and normal in one patient (data missing in one). One had *Staphylococcus lugdunensis* in 5/5 cultures of periprosthetic synovial tissue biopsies and protocol samples. Another one was re-classified based on cultures of *E. faecalis* from periprosthetic synovial tissue biopsies (5/5), joint fluid and sonication fluid. A third was positive with cultures of *Staphylococcus capitis* in 3/5 periprosthetic synovial tissue biopsies. Cultures from the fourth patient yielded *Staphylococcus epidermidis* from synovial tissue biopsies (5/5) and protocol samples.

### 16S *rRNA* gene amplification (Appendix 3)

Periprosthetic synovial tissue biopsies were negative by molecular testing. Joint fluid was positive in two patients: One patient in group 3 with *S. lugdunensis* PJI (case 1) and one patient in group 1 with *Finegoldia magna* in both joint fluid and a bone biopsy (case 16).

Sonication fluid polymerase chain reaction (PCR) was positive in three patients in group 3, all being concordant with cultures of synovial fluid and tissue biopsies (cases 1, 2 and 4). Bone tissue biopsies were positive in two patients re-classified as AF in group 1 (cases 16 and 18). Molecular analysis of swabs obtained from the implant surface was positive in four patients of which one was concordant with synovial tissue culture (group 3, case 4).

## Discussion

In the present prospective study, we report a cohort of patients undergoing revision and investigated for AF according to a diagnostic algorithm and undergoing revision surgery [23]. We evaluated results for 71 patients undergoing revision surgery for AF after THA and TKA within a larger study addressing the diagnosis of prosthesis-related infection and pain. There is no gold standard for diagnosis of PJI, and therefore we maintained the culturing of five periprosthetic synovial tissue biopsies as the reference method. This has been the routine for approximately 20 years in our orthopaedic department [13], and on this background, we judged cautiously the results obtained with new sample types, modified culture methods, and molecular tests.

We found five of 72 cases with PJI not recognized prior to surgery. Three cases were detected with our reference standard, i.e. periprosthetic synovial tissues biopsies cultured for 6 days. Two additional cases were found by extended incubation (14 days) of joint fluid and cultivation of sonication fluid within the framework of the project. Combining standard and project diagnostics, complete negative results were obtained for 52 patients (53 procedures, 73.6%). Twelve patients had spurious positive findings on standard and project diagnostics deemed clinically insignificant (see Appendix 3).

WBC and CRP did not differ between the three groups. We do not consider the CRP difference between group 1 and 3 clinically relevant, since there are only three observations in group 3. Our findings with normal BMI (< 25) and not having positive periprosthetic synovial tissue biopsy cultures was in agreement with others [26].

The six patients who had multimodal nuclear imaging were not excluded as they still met criteria for revision surgery for AF after imaging. Multimodal nuclear imaging is not validated as a part of evaluating AF and was not a part of the diagnostic work-up of AF, nevertheless clinical acumen supported revision surgery. Culturing and 16S *rRNA* amplification test were negative in all cases.

We obtained concordant results for cultures of synovial tissue biopsies and sonication fluid in three PJI patients. Results discordant with synovial tissue biopsies were found in three patients. One was re-classified PJI-indeterminable (group 1 case 11). The two last patients were re-classified to confirmed AF (group 2 case 7 and group 1 case 14).

Relatively few studies of patients with a failing THA or TKA have focused entirely on AF and applied a broad range of laboratory diagnostic methods [18, 20]. Ribera et al. [19] studied 89 patients with AF as defined by clinical and radiological criteria and absence of local signs or symptoms of infection. PJI was defined by histopathology, intraoperative purulence, and at least two cultures with the same pathogen. Disregarding results from sonication fluid, 13% (12/89) were diagnosed with occult PJI. Concordance between cultures from tissue samples and sonication fluid was 75% (9/12).

Trampuz et al. [27] analyzed cultures of synovial tissue biopsies and sonication fluid in 331 patients undergoing revision for PJI or AF [27]. AF (*n* = 252) was a postoperative diagnosis defined by prosthetic failure and absence of PJI criteria which mainly were overt signs of infection and positive histopathology. Twenty-one patients with a diagnosis of AF had a single positive tissue culture and two had two or more positive cultures. Culturing of sonication fluid was positive in three cases (1.2%) although tissue culture was negative. Direct comparison with our study is difficult, but unmistakably positive cultures seemed to be rare in patients undergoing revision for AF.

Fernandez-Sampedro et al. [20] compared cultures from sonication fluid and periprosthetic tissue biopsies in patients undergoing revision for AF (*n* = 198). Pre- and intraoperative diagnoses were based on strict criteria including radiological and radionuclide imaging, the absence of clinical signs of infection, and normal inflammatory markers. Similar to Trampuz et al., postoperative diagnoses of AF were established by the absence of PJI. Furthermore, isolation of a microorganism from sonication fluid in an amount of at least 20 colony forming units or a single positive culture of *S. aureus* or *S. lugdunensis* were a part of the definition. Based on these criteria a diagnosis of AF was made in 174 cases (88%). Disregarding histopathology, 16 patients (8.1%) had occult PJI based on positive cultures from periprosthetic tissue and sonication fluid. Positive culture of sonication fluid, but negative cultures of periprosthetic tissue were found in 5 cases (2.5%). Compared with our study the frequency of PJI in Spanish patients undergoing revision for AF was notably higher than in our study (12, 95% CI: 8–17%). Previous antibiotic therapy may mask PJI at least for a period of time, and it is noteworthy that few Danish patients had been treated incidentally for another reason. The Spanish study does not provide this information, but it is noteworthy that antibiotics are available without prescription in Spain [28]. Antibiotics are a prescription drug in Denmark.

Periprosthetic tissue and project samples were examined with 16S *rRNA* gene PCR and amplicon sequencing in our study. Three of five patients re-classified to PJI had confirmatory PCR findings for sonication fluid. Of three patients previously treated with antibiotics, none had positive findings by PCR in the collective set of standard and protocol samples. Gomez et al. [29] evaluated 16S *rRNA* gene PCR performed with sonication fluid in 231 patients with AF diagnosed postoperatively similar to Trampuz et al. Three patients had positive PCR, but negative sonication fluid and tissue culture. Two of these patients had prior antibiotic treatment. Information on the indication for revision and re-classification was not reported. Molecular methods have theoretical advantages for detecting microbial pathogens in association with prosthetic failure. Currently, this attracts much interest, and our findings suggest that experienced surgeons can distinguish PJI and AF from each other with high accuracy and we found little support for the hypothesis that occult infection is a frequent cause of AF.

Results from the different studies including this performed under the PRIS project did not support a significant value of routine molecular tests of intraoperative tissue in patients suspected of AF. Details from 16S *rRNA* in this study have been published elsewhere [23].

A major strength of our study was the algorithm rooted in a clinical setting. The strict protocol of intraoperative tissue collection and the post-operative laboratory analysis led to an unbiased comparison of culture-dependent and independent methods. We feel confident that missing data from inclusion on patient history and during follow-up was controlled by linkage to the medical records, laboratory and microbiological database. All patients are registered in the hospital and regional administrative data system allowing for merging and linking to the clinical databases. Additionally a close collaboration between the participating clinical specialties was valuable for patient management.

Our study has a number of limitations. The inclusion of patients followed a pragmatic study design. Distinguishing between AF and PJI was done by a senior Orthopaedic clinician and followed the existing practice in our Institute. This included patient history, physical examination and X-ray. Thus the pre-operative identification of AF, including the six patients with previous multimodal nuclear imaging, was predominantly a clinical decision. These issues reflect the challenges in this field.

Furthermore the low number of patients and consequently few infected cases complicates conclusion. Histopathology and biochemical analysis of synovial fluid were not part of the algorithm and are not used in Denmark on a routine basis. This hampered comparisons with other European studies. Biochemical analyses were not specified as inclusion criteria, and CRP values were incomplete in 13 patients. Nevertheless, three of five patients with unrecognized PJI had elevated CRP values. These three patients were not excluded, as it was not deemed clinically relevant at inclusion. Several studies of the diagnostic utility of inflammatory markers have been published recently [30, 31]. Biochemistry including CRP should be part of pre-operative evaluation, however it cannot stand alone. In other studies it is reported as a minor criteria for PJI [14]. FDG PET/CT scans was not a planned diagnostic method for AF, nevertheless clinical acumen supported revision in 6 cases.

## Conclusion

In conclusion, our results showed a low incidence of PJI in cases suspected of AF pre-operatively in patients with THA or TKA. Experimental specimen types and prolonged cultures were conducive to detection of two of five PJI cases unrecognized prior to surgery. We were able to control for missed PJI diagnoses through access to health databases within the North Denmark Region. One patient presented with PJI deemed unrelated to the failure of the previous arthroplasty.

A structured approach is advisable to identify unrecognized PJI in revision for AF. The algorithm served as a useful tool. However, clinical judgment should not be outweighed in the pre-operative decision-making process. Extended incubation improved diagnostics in accordance with the existing literature. Sonication of retrieved components did not provide additional information in this study. A previous paper with a methodological aim analyzed in depth the differential contribution of specimen types, extended incubation of cultures, and 16S rRNA sequencing from this study.

## Data Availability

The datasets generated and/or analysed during the current study are not publicly available due to privacy and copyright, but are available from the corresponding author on reasonable request.

## Appendix 1

If a clinical suspicion of PJI was present and less than 8 weeks had passed since primary surgery and/or clinical signs of a haematogenous infection was present; revision for acute infection was scheduled.

If more than 8 weeks had passed or clinical evaluation did not reveal the underlying cause, further evaluation was made with multi-modal radionucleid imaging in order to characterize the chronic problem. Imaging comprised ^99m^Tc – HDP SPECT/CT bone scan, dual ^111^In-labeled white blood cells SPECT/CT scan combined with ^99m^Tc-nannocol bone marrow scan, and ^18^F-FDG PET/CT scan on three consecutive days followed by a multidisciplinary conference. A subset of patients with focal findings were evaluated with a bioptic procedure or scheduled for revision surgery for a chronic infection. Intra-operative diagnostic setup was identical to revision for acute infection and aseptic failure. If clinical suspicion of PJI was not present, patients were diagnosed with chronic pain. This group was followed for 12 months (median) for change in the clinical status.

## Appendix 2

Protocol for samples obtained during revision surgery:

Project samples: PS.

Standard tissue samples: STS.

In order to minimize contamination, joint fluid was aspirated once the joint was exposed, but prior to incision of the capsule (PS). Once the capsule was incised, three swabs were taken from the surface of the implant, in THA from the femoral head and in TKA from the femoral component. Immediately afterwards three synovial biopsies were taken from the vicinity of the prosthesis (PS). Next in line were 5 synovial tissue biopsies (STS) taken from the same area as the 3 samples. If only the polyethylene insert was exchanged, 3 periprosthetic bone biopsies were taken with a trocar from the bone-joint interface (PS). If other components were removed, bone sampling was withheld and taken from the exposed bone surface. All removed components (PS) were collected and placed directly in an assigned container by the surgeon.

Sampling was done with sterile disposable utensils separate for each sample. Intraoperative antibiotics were withheld until all samples were collected. Routine antibiotic treatment was intravenous cefuroxime until culture results were available (negative results were informed on day 6).

## Appendix 3

Positive culture and molecular findings from 19 surgical procedures. Aseptic failure (AF), Prosthetic joint infection (PJI), Prosthetic joint infection-indeterminable (PJI-indeterminable.)

**Group 3**: 3–5 positive periprosthetic synovial tissue cultures.

**Table:**

	Periprosthetic tissue		Joint fluid		Sonication		Bone		Swab	
Case nr./Re-classification from AF	Culture	PCR	Culture	PCR	Culture	PCR	Culture	PCR	Culture	PCR
1 PJI	*S. lugdunensis* 5/5	Neg	*S.* *lugdunensis*	*S.* *lugdunensis*	*S. lugdunensis*	*S. lugdunensis*	Neg	Neg	*S.* *lugdunensis*	Neg
2 PJI	*E. faecalis* 5/5	Ne	*E. faecalis*	Neg	*E. faecalis*	*E. faecalis*	Neg	Neg	Neg	Neg
3 PJI	*S. capitis* 3/5	Neg	Neg	Neg	Neg	Neg	Neg	Neg	Neg	Neg
4 PJI	*S. epidermidis* 5/5	Neg	*S. epidermidis*	___	*S. epidermidis*	*S. epidermidis*	*S. epidermidis*	Neg	*S. epidermidis*	*S. epidermidis*

**Group 2**: 1–2 positive periprosthetic synovial tissue cultures

**Table:**

	Periprosthetic tissue		Joint fluid		Sonication		Bone		Swab	
Case nr./Re-classification from AF	Culture	PCR	Culture	PCR	Culture	PCR	Culture	PCR	Culture	PCR
5 PJI	*P. acnes* (2/5)	Neg	*P. acnes*	Neg	Neg	Neg	Neg	Neg	Neg	Neg
6 PJI-indeter.	*P. acnes* (1/5)	Neg	*P. acnes*	Neg	Neg	Neg	Neg	Neg	Neg	Neg
7 AF	*S. capitis* (1/5)	___	Neg	Neg	*S. caprae*	Neg	Neg	___	Neg	___
8 AF	*Staphylococcus* sp*.* (1/5)	Neg	Neg	Neg	Neg	Neg	Neg	Neg	Neg	Neg
9 AF	*P. acnes* (1/5)	Neg	Neg	Neg	Neg	Neg	Neg	Neg	___	___
10 AF	CoNS (2/5)	Neg	Neg	Neg	Neg	Neg	Neg	Neg	Neg	Neg

**Group 1**: 0 positive periprosthetic synovial tissue cultures

**Table:**

	Periprosthetic tissue		Joint fluid		Sonication		Bone		Swab	
Case nr./Re-classification from AF	Culture	PCR	Culture	PCR	Culture	PCR	Culture	PCR	Culture	PCR
11 PJI-indeter.	Neg	Neg	*E. coli*	Neg	*E. coli*	Neg	Neg	Neg	Neg	Neg
12 AF	Neg	Neg	Neg	Neg	Neg	Neg	Neg	Neg	Neg	*Staphylococcus* sp*.*
13 AF	Neg	Neg	Neg	Neg	Neg	Neg	Neg	Neg	Neg	*F. magna*
14 AF	Neg	Neg	Neg	Neg	*P. avidum*	Neg	Neg	Neg	Neg	Neg
15 AF	Neg	Neg	___	Neg	Neg	Neg	___	___	Neg	*F. magna*
16 AF	Neg	Neg	Neg	*F. magna*	Neg	Neg	Neg	*F. magna*	Neg	Neg
17 AF	Neg	Neg	*P. acnes*	Neg	Neg	Neg	Neg	Neg	Neg	Neg
18 AF	Neg	Neg	Neg	Neg	Neg	Neg	Neg	*Clostridium* sp*.*	Neg	Neg
19 AF	Neg	Neg	Neg	Neg	Neg	Neg	Neg	Neg	*Aerob Gram-positive rods*	Neg

**Textbox 1**. Definition of re-classification based on the laboratory test of standard tissue samples

**Table:**

**PJI** ≥3 positive cultures with the same microorganism(s) **PJI-indeterminable** 2 positive cultures with the same microorganism(s) **Confirmed AF** Absence of above criteria	

## Abbreviations

AF: Aseptic failure
CRP: C-reactive protein
DHR: Danish Hip Arthroplasty Registries
DKR: Danish Knee Arthroplasty Registries
FDG: Fluor-deoxy-glucose
PCR: Polymerase chain reaction
PJI: Prosthetic joint infection
THA: Total hip arthroplasty
TKA: Total knee arthroplasty
WBC: White blood cell
